# Association Between Preexisting Heart Failure With Reduced Ejection Fraction and Fluid Administration Among Patients With Sepsis

**DOI:** 10.1001/jamanetworkopen.2022.35331

**Published:** 2022-10-07

**Authors:** Rachel E. Powell, Jason N. Kennedy, Mourad H. Senussi, Ian J. Barbash, Christopher W. Seymour

**Affiliations:** 1 Division of Pulmonary, Allergy, and Critical Care Medicine, University of Pittsburgh Medical Center, Pittsburgh, Pennsylvania; 2 Department of Critical Care Medicine, University of Pittsburgh Medical Center, Pittsburgh, Pennsylvania; 3 Clinical Research, Investigation, and Systems Modeling of Acute Illness Center, Pittsburgh, Pennsylvania; 4 Division of Cardiology and Critical Care Medicine, Baylor College of Medicine, Houston, Texas; 5 Department of Emergency Medicine, University of Pittsburgh, Pittsburgh, Pennsylvania

## Abstract

**Importance:**

Intravenous fluid administration is recommended to improve outcomes for patients with septic shock. However, there are few data on fluid administration for patients with preexisting heart failure with reduced ejection fraction (HFrEF).

**Objective:**

To evaluate the association between preexisting HFrEF, guideline-recommended intravenous fluid resuscitation, and mortality among patients with community-acquired sepsis and septic shock.

**Design, Setting, and Participants:**

A cohort study was conducted of adult patients hospitalized in an integrated health care system from January 1, 2013, to December 31, 2015, with community-acquired sepsis and preexisting assessment of cardiac function. Follow-up occurred through July 1, 2016. Data analyses were performed from November 1, 2020, to August 8, 2022.

**Exposures:**

Preexisting heart failure with reduced ejection fraction (≤40%) measured by transthoracic echocardiogram within 1 year prior to hospitalization for sepsis.

**Main Outcomes and Measures:**

Multivariable models were adjusted for patient factors and sepsis severity and clustered at the hospital level to generate adjusted odds ratios (aORs) and 95% CIs. The primary outcome was the administration of 30 mL/kg of intravenous fluid within 6 hours of sepsis onset. Secondary outcomes included in-hospital mortality, intensive care unit admission, rate of invasive mechanical ventilation, and administration of vasoactive medications.

**Results:**

Of 5278 patients with sepsis (2673 men [51%]; median age, 70 years [IQR, 60-81 years]; 4349 White patients [82%]; median Sequential Organ Failure Assessment score, 4 [IQR, 3-5]), 884 (17%) had preexisting HFrEF, and 2291 (43%) met criteria for septic shock. Patients with septic shock and HFrEF were less likely to receive guideline-recommended intravenous fluid than those with septic shock without HFrEF (96 of 380 [25%] vs 699 of 1911 [37%]; *P* < .001), but in-hospital mortality was similar (47 of 380 [12%] vs 244 of 1911 [13%]; *P* = .83). In multivariable models, HFrEF was associated with a decreased risk-adjusted odds of receiving 30 mL/kg of intravenous fluid within the first 6 hours of sepsis onset (aOR, 0.63; 95% CI, 0.47-0.85; *P* = .002). The risk-adjusted mortality was not significantly different among patients with HFrEF (aOR, 0.92; 95% CI, 0.69-1.24; *P* = .59) compared with those without, and there was no interaction with intravenous fluid volume (aOR, 1.00; 95% CI, 0.98-1.03; *P* = .72).

**Conclusions and Relevance:**

The results of this cohort study of patients with community-acquired septic shock suggest that preexisting HFrEF was common and was associated with reduced odds of receiving guideline-recommended intravenous fluids.

## Key Points

**Question:**

What is the association between preexisting heart failure with reduced ejection fraction (HFrEF) and appropriate fluid resuscitation among patients with sepsis?

**Findings:**

In this cohort study of 5278 patients with community-onset sepsis, 884 (17%) had preexisting HFrEF, which was associated with a lower risk-adjusted odds of receiving 30 mL/kg of intravenous fluids within 6 hours of sepsis onset. However, there was no association between HFrEF and in-hospital mortality.

**Meaning:**

The results of this study suggest that preexisting HFrEF is common among patients with septic shock and is associated with reduced odds of receiving guideline-recommended intravenous fluids.

## Teaser

This cohort study evaluates the association between preexisting heart failure with reduced ejection fraction, guideline-recommended intravenous fluid resuscitation, and mortality among patients with community-acquired sepsis and septic shock.

## Introduction

Sepsis is a life-threatening organ dysfunction caused by a dysregulated host response to infection, and more than 1 in 5 patients admitted with sepsis die during their hospital stay.^1,2,3^ Intravenous fluid administration is a key component of clinical practice guidelines and federally mandated performance benchmarks for the management of sepsis and septic shock.^4,5^ However, there is little evidence from randomized clinical trials to support a strategy of aggressive intravenous fluid administration, and there is increasing concern about the downstream harms of fluid overload.^6^

Guidelines recently downgraded the recommendation for a 30-mL/kg intravenous fluid bolus among all patients with sepsis-induced hypoperfusion from a strong to a weak recommendation.^7^ However, controversy remains because aggressive intravenous fluid administration is a component of the Centers for Medicare & Medicaid Services (CMS) SEP-1 bundle and is widely used in clinical practice.^8^ Clinicians are particularly concerned about the potential harms of empirical fluid administration to patients with underlying heart failure with reduced ejection fraction (HFrEF), who may be at higher risk of complications.^9,10,11,12,13,14,15,16,17,18^ A greater understanding of the epidemiology of preexisting HFrEF prior to sepsis and its association with outcomes is needed to balance the pros and cons of fluid resuscitation for these patients at high risk. To address these knowledge gaps, in a multicenter cohort study, we explored the epidemiology of HFrEF diagnosed prior to hospitalization for sepsis and its risk-adjusted association with guideline-recommended intravenous fluid administration and outcome during emergency care of septic shock.

## Methods

The study was approved by the University of Pittsburgh Human Research Protection Office. The data were obtained under a waiver of informed consent because the data were deidentified and with authorization under the Health Insurance Portability and Accountability Act. All reporting was in accordance with the Strengthening the Reporting of Observational Studies in Epidemiology (STROBE) reporting guideline for cohort studies.^19^

### Data Sources

We used electronic health record data (Cerner Co) from encounters at 11 community and academic hospitals at the University of Pittsburgh Medical Center (UPMC) between January 1, 2013, and December 31, 2015.^1,20^ Follow-up occurred through July 1, 2016. These data included patient demographic characteristics, vital signs, laboratory test values, medication administration, microbiology test results, organ support, length of stay, and in-hospital mortality. Patient-reported race and ethnicity were derived from the UPMC registration system data using fixed categories consistent with the CMS electronic health record meaningful use data set. Comorbid conditions were identified using hospitalization diagnosis *International Classification of Diseases, Ninth Revision* codes. For descriptions of illness acuity, the most abnormal vital sign and laboratory values were abstracted within 6 hours of sepsis onset.

Structured echocardiographic data were obtained from prior inpatient (Cerner Co) and outpatient (Epic Systems Co) transthoracic echocardiography reports. Parameters electronically abstracted included left ventricular ejection fraction (LVEF), presence and severity of right ventricular (RV) dysfunction, estimated pulmonary artery systolic pressure, and the presence and severity of valvular abnormalities. Data from transesophageal or point-of-care echocardiograms were excluded.

### Study Design and Patients

We evaluated patients with community-onset sepsis. Inclusion criteria were (1) adults (aged ≥18 years) hospitalized for more than 24 hours, (2) meeting Sepsis-3^1^ criteria within the first 6 hours of presentation, and (3) with transthoracic echocardiography performed at UPMC between 1 and 365 days prior to their presentation with sepsis.

To identify patients meeting Sepsis-3 criteria in the electronic health record, patients had to have met the following criteria within 6 hours of arrival to the emergency department: (1) evidence of suspected infection and (2) presence of organ dysfunction. Evidence of suspected infection was defined as the combination of administration of antibiotics (oral or parenteral) and a body fluid culture specimen obtained. The presence of organ dysfunction was defined as a Sequential Organ Failure Assessment (SOFA) score^21^ of 2 or higher. To understand the performance of guideline-recommended care, we identified patients as having septic shock based on the CMS SEP-1 criteria for hypoperfusion: (1) mean arterial pressure of 65 mm Hg or lower within the first 6 hours, (2) serum lactate level of 4 mmol/L or higher (to convert to milligrams per deciliter, divide by 0.111) within the first 6 hours, or (3) requirement of vasopressors within the first 48 hours. Patients were excluded if they were transferred from non-UPMC institutions.

The primary exposure was HFrEF. Patients were classified as having HFrEF if they met the 2013 American Heart Association (AHA) criterion of ejection fraction (EF) of 40% or less on transthoracic echocardiogram prior to hospitalization for sepsis.^22^ Heart failure with preserved ejection fraction was not examined owing to concerns about inaccurate patient classification using electronic health record data alone.

### Study Outcomes

The primary outcome was the administration of 30 mL/kg or more of intravenous fluids within the first 6 hours of presentation, as recommended by the CMS SEP-1 bundle. Intravenous fluids included any crystalloid solution (eg, 0.9%, sodium chloride; lactated Ringer solution; and commercial balanced crystalloid solution [Plasma-Lyte; Baxter International Inc]). Secondary outcomes included in-hospital mortality, intensive care unit admission, rate of invasive mechanical ventilation, and administration of vasoactive medications.

### Statistical Analysis

Statistical analyses were performed from November 1, 2020, to August 8, 2022. We addressed 3 knowledge gaps in this study: (1) the epidemiology of preexisting HFrEF among patients hospitalized for sepsis, (2) the risk-adjusted association between preexisting HFrEF and guideline-recommended intravenous fluid administration during emergency care, and (3) whether preexisting HFrEF modified the risk-adjusted association between guideline-recommended intravenous fluid administration and in-hospital mortality. The first descriptive analysis was conducted among all patients meeting Sepsis-3 criteria. The risk-adjusted analyses were restricted to patients with septic shock, the target population of the CMS SEP-1 bundle.

We compared baseline characteristics and outcomes of patients with or without HFrEF. Categorical data were presented as number and percentage and compared with χ^2^ testing. Normally distributed continuous data were presented as mean (SD) values and compared using *t* testing, while nonnormally distributed continuous data were presented as median (IQR) values and were compared using Kruskal-Wallis testing.

Prior to modeling, we assessed variable distributions, missingness, and correlation. Log transformation was used for nonsymmetrically distributed data. In the primary analysis, we used multivariable logistic regression with robust SEs to quantify the risk-adjusted association between HFrEF and the receipt of 30 mL/kg of intravenous fluid within the first 6 hours among patients meeting criteria for septic shock. In secondary analyses, we used multivariable logistic regression with robust SEs to quantify the risk-adjusted association between HFrEF and in-hospital mortality, and we tested for effect modification using the Wald test for interaction between HFrEF and the volume of fluid administered within the first 6 hours.

Model covariates for the primary and secondary models were chosen a priori based on factors known to be associated with fluid administration and sepsis-specific outcomes, including age, sex, race and ethnicity (Black, White, other [American Indian or Alaska Native, Asian, Chinese, Filipino, Hawaiian or Other Pacific Islander, Middle Eastern, Native American, not specified, or Pacific Islander]), Elixhauser Comorbidity Index (range, 0-31; with higher score indicating greater comorbidity burden), and presenting SOFA score (range, 0-24).^21^ All models were clustered at the hospital level using a sandwich estimator for variance to account for variations in practice patterns at the different sites. Analyses were performed in Stata, version 17.0 (StataCorp LLC) and PRISM 9.0 (GraphPad Software LLC). All *P* values were from 2-sided tests, and results were deemed statistically significant at *P* < .05.

We performed multiple sensitivity analyses to understand the robustness of the results. First, we quantified the strength required for a hypothetical unmeasured confounder to negate the primary analysis association between HFrEF and fluid administration using the E-value.^23^ Second, because intravenous fluids are administered not just to patients with septic shock, we evaluated models that included patients with less severe sepsis who did not meet CMS SEP-1 criteria for septic shock. Third, because of the uncertainty of the timing of incident sepsis causing sepsis-associated myocardial dysfunction, we evaluated models using only patients with echocardiographic studies more than 1 month prior to hospitalization. Fourth, because RV dysfunction could potentially confound the associations between HFrEF and intravenous fluid administration or mortality, we included RV dysfunction as a covariate in our primary and secondary models. Fifth, we repeated our primary and secondary analyses using continuous LVEF as the primary exposure of interest instead of HFrEF, and we used a fractional polynomial assessment for nonlinear associations.^24^

## Results

### Patients

**Figure 1.**
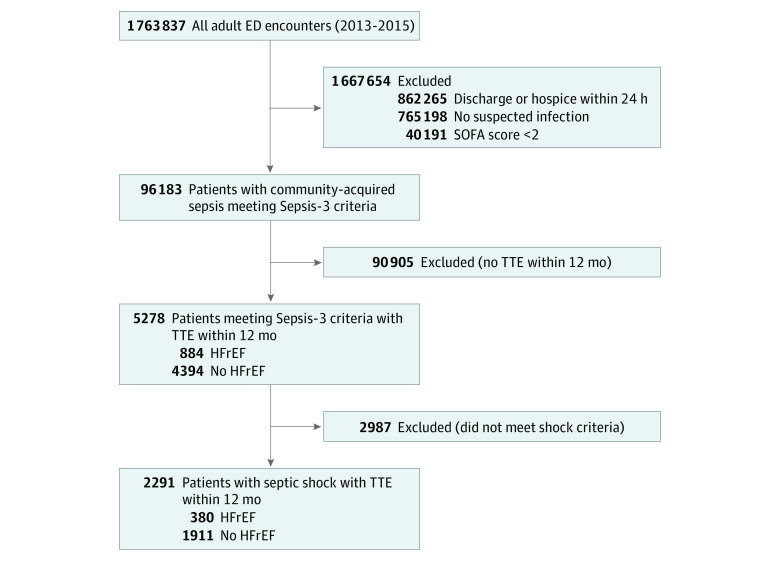
Flow Diagram of Patients in the Cohort ED indicates emergency department; HFrEF, heart failure with reduced ejection fraction; SOFA, Sequential Organ Failure Assessment; and TTE, transthoracic echocardiography.

We identified 96 183 unique patients who met Sepsis-3 criteria within 6 hours of presentation to the emergency department, of whom 5278 (6%; 2673 men [51%]; median age, 70 years [IQR, 60-81 years]; 4349 White patients [82%]; median SOFA score, 4 [IQR, 3-5]) had a transthoracic echocardiogram within 1 year prior to arrival (Figure 1). Of these, 884 patients (17%) had HFrEF, and 2291 (43%) met criteria for septic shock (Table 1). The distribution of LVEF was similar among patients with sepsis and septic shock (eFigure in the Supplement). The mean (SD) Elixhauser Comorbidity Index was 5.5 (2.2), and the median serum lactate level was 1.8 mmol/L (IQR, 1.2-2.9 mmol/L). Overall, 712 patients (14%) required vasoactive medications, and 449 (9%) died during hospitalization (eTable 1 in the Supplement).

**Table 1.** Baseline Characteristics of Patients (N = 5278)

Characteristic	Patients, No. (%)					
	All meeting Sepsis-3 criteria		Meeting Sepsis-3 criteria without shock		Septic shock	
	Normal EF (n = 4394)	HFrEF (n = 884)	Normal EF (n = 2483)	HFrEF (n = 504)	Normal EF (n = 1911)	HFrEF (n = 380)
Age, median (IQR), y	70 (59-81)	73 (62-82)	70 (59-81)	72.5 (62-82)	70 (60-81)	73 (63-82)
Sex						
Male	2106 (48)	567 (64)	1260 (51)	340 (68)	846 (44)	227 (60)
Race and ethnicity						
Black	656 (15)	177 (20)	397 (16)	102 (20)	259 (14)	75 (20)
White	3666 (83)	683 (77)	2052 (83)	385 (76)	1614 (85)	298 (78)
Other^a^	72 (2)	24 (3)	34 (1)	17 (3)	38 (2)	7 (2)
Elixhauser Comorbidity Index, mean (SD)^b^	5.4 (2.2)	5.7 (2.2)	5.3 (2.2)	5.6 (2.2)	5.6 (2.2)	5.8 (2.2)
Clinical characteristic^c^						
SOFA score, median (IQR)^d^	4 (2-5)	4 (3-5)	3 (2-4)	3 (2-5)	4 (3-6)	4 (3-6)
Mean arterial pressure, mean (SD), mm Hg	69.3 (15.7)	68.9 (15.9)	79.5 (11.4)	79.0 (10.9)	56.2 (9.4)	55.6 (10.8)
Oxygen saturation, median (IQR), %	93 (89-95)	93 (90-95)	93 (89-95)	93 (91-95)	93 (88-95)	93 (89-95)
Lactate, median (IQR), mmol/L	1.8 (1.2-2.9)	2.0 (1.3-2.8)	1.5 (1.1-2.1)	1.7 (1.1-2.3)	2.1 (1.3-3.5)	2.1 (1.4-3.6)
Platelet count, median (IQR), 10^3^/µL	213 (144-290)	205 (151-277)	211 (145-285)	204 (149-274)	219 (145-299)	208 (155-286)
Creatinine, median (IQR), mg/dL	1.4 (0.9-2.3)	1.7 (1.2-2.9)	1.3 (0.9-2.2)	1.7 (1.2-2.8)	1.5 (1.0-2.4)	1.7 (1.2-3.0)
SEP-1 process measures						
Antibiotics given within 3 h	3018 (69)	620 (70)	1658 (67)	347 (69)	1360 (72)	273 (72)
Lactate checked within 3 h	1840 (42)	352 (40)	737 (30)	146 (29)	1103 (58)	206 (54)
30 mL/kg intravenous fluids administered within 6 h	885 (20)	113 (13)	186 (8)	17 (3)	699 (37)	96 (25)
Source of infection						
Bloodstream	584 (13)	125 (14)	249 (10)	60 (12)	335 (18)	65 (17)
Urine	838 (19)	145 (16)	447 (18)	91 (18)	391 (21)	54 (14)
Pulmonary	403 (9)	66 (8)	189 (8)	19 (4)	214 (11)	47 (12)
Unknown	2175 (50)	461 (52)	1382 (56)	285 (57)	793 (42)	176 (46)
Other	394 (9)	87 (10)	216 (9)	49 (10)	178 (9)	38 (10)
Echocardiographic parameter						
LVEF, median (IQR), %	55 (55-60)	30 (25-35.5)	55 (55-60)	30 (20-38)	55 (55-60)	30 (20-35)
RV dysfunction	240 (6)	161 (18)	120 (5)	87 (19)	120 (6)	74 (20)
Estimated pulmonary artery systolic pressure, median (IQR), mm Hg	39 (30-48)	41 (33-51)	39 (30-48.5)	43 (33-53)	39 (30-48)	40 (31-49)
Severe mitral valve regurgitation	245 (6)	159 (18)	146 (6)	97 (19)	99 (5)	62 (16)
Severe aortic valve regurgitation	72 (2)	24 (3)	50 (2)	13 (3)	22 (1)	11 (3)

Abbreviations: EF, ejection fraction; HFrEF, heart failure with reduced ejection fraction; LVEF, left ventricular ejection fraction; RV, right ventricular; SOFA, Sequential Organ Failure Assessment.

SI conversion factors: To convert lactate to milligrams per deciliter, divide by 0.111; platelets to 10^9^ per liter, multiply by 1.0; and creatinine to micromoles per liter, multiply by 88.4.

^a^ American Indian or Alaska Native, Asian, Chinese, Filipino, Hawaiian or Other Pacific Islander, Middle Eastern, Native American, not specified, or Pacific Islander.

^b^ A method of categorizing the comorbid conditions of patients based on the *International Classification of Diseases, Ninth Revision* diagnosis codes found in administrative data (range, 0-31; with higher score indicating greater comorbidity burden).

^c^ Corresponds to minimum or maximum value, as appropriate, within the first 6 hours.

^d^ SOFA score corresponds to the severity of organ dysfunction within 6 hours of sepsis onset, reflecting 6 organ systems (cardiovascular, hepatic, hematologic, respiratory, neurologic, kidney), each with a score range of 0 to 4 points, with a total score range of 0 to 24 points.

### Epidemiology of Preexisting HFrEF and Intravenous Fluid Administration

Among 5278 eligible patients with sepsis, those with HFrEF were more commonly male (567 of 884 [64.1%] vs 2106 of 4394 [47.9%]; *P* < .001), Black (177 of 884 [20.0%] vs 656 of 4394 [14.9%]; *P* < .001), and presented with elevated serum creatinine levels (median, 1.7 mg/dL [IQR 1.2-2.9 mg/dL] vs 1.4 mg/dL [IQR, 0.9-2.3 mg/dL] [to convert to micromoles per liter, multiply by 88.4]; *P* < .001) compared with those with normal EF (Table 1). There were no significant differences between patients with HFrEF and those with normal EF in maximum SOFA score within 6 hours (median, 4 [IQR, 3-5] vs 4 [IQR, 2-5]; *P* = .44) or serum lactate level (median, 2.0 mmol/L [IQR, 1.3-2.8 mmol/L] vs 1.8 mmol/L [IQR, 1.2-2.9 mmol/L]; *P* = .26). On the baseline transthoracic echocardiogram, patients with HFrEF had more RV dysfunction (161 of 884 [18%] vs 240 of 4394 [6%]; *P* < .001) and severe mitral valve regurgitation (159 of 884 [18%] vs 245 of 4394 [6%]; *P* < .001) than did patients with normal EF.

A total of 795 of 2291 patients (35%) with septic shock received at least 30 mL/kg of intravenous fluids within 6 hours of sepsis onset (eTable 1 in the Supplement). The proportion of patients receiving 30 mL/kg of intravenous fluids was significantly lower among patients with septic shock and HFrEF than among patients with septic shock and normal EF (96 of 380 [25%] vs 699 of 1911 [37%]; *P* < .001). On average, patients with septic shock and HFrEF received less fluid total at 6 hours than patients with septic shock and normal EF (15.6 mL/kg [IQR, 5.3-30.2 mL/kg] vs 20.5 mL/kg [IQR, 7.7-39.4 mL/kg]; *P* < .001). There was no difference in the proportion of patients with septic shock receiving broad-spectrum antibiotics within 3 hours (273 of 380 [72%] with HFrEF vs 1360 of 1911 [71%] with normal EF; *P* = .79) or having their serum lactate level measured within 3 hours (206 of 380 [54%] with HFrEF vs 1103 of 1911 [58%] with normal EF; *P* = .21) (Table 1).

In unadjusted comparisons, the in-hospital mortality of patients with septic shock was similar for those with or without HFrEF (47 of 380 [12%] vs 244 of 1911 [13%]; *P* = .83) (eTable 1 in the Supplement). There was no difference between patients with and patients without HFrEF in the median duration of invasive mechanical ventilation (4 days [IQR, 3-10 days] vs 5 days [IQR, 3-10 days]; *P* = .56), receipt of vasoactive medications (121 of 380 [32%] vs 591 of 1911 [31%]; *P* = .72), intensive care unit admission (304 of 380 [80%] vs 1495 of 1911 [78%]; *P* = .44), or median hospital length of stay (6.9 days [IQR, 4.5-11.5 days] vs 7.1 days [IQR, 4.5-11.5 days]; *P* = .36).

### Risk-Adjusted Association Between Preexisting HFrEF and Guideline-Recommended Intravenous Fluid Administration

**Figure 2.**
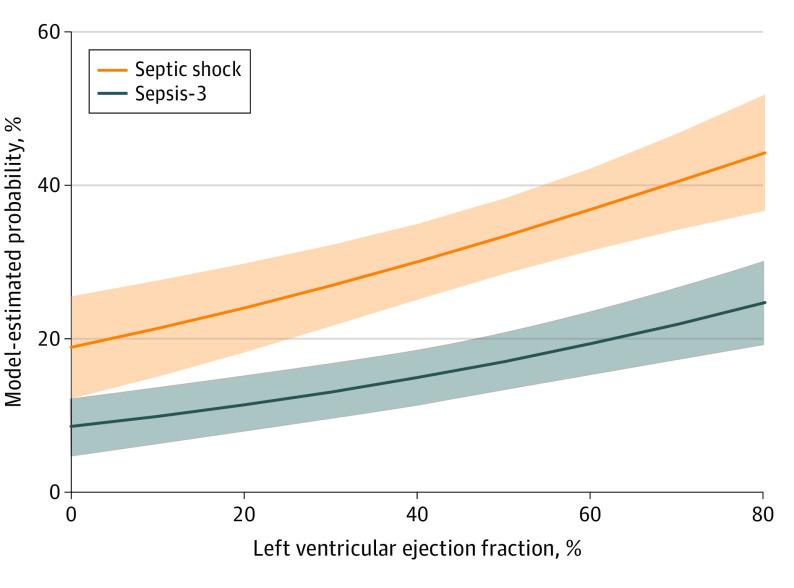
Estimated Probability of Intravenous Fluid Administration by Left Ventricular Ejection Fraction Measured Within 1 Year Prior to Hospitalization for Sepsis Model-estimated probability of receiving 30 mL/kg of intravenous fluids among patients meeting septic shock criteria or Sepsis-3 criteria. Shaded areas indicate 95% CIs.

**Figure 3.**
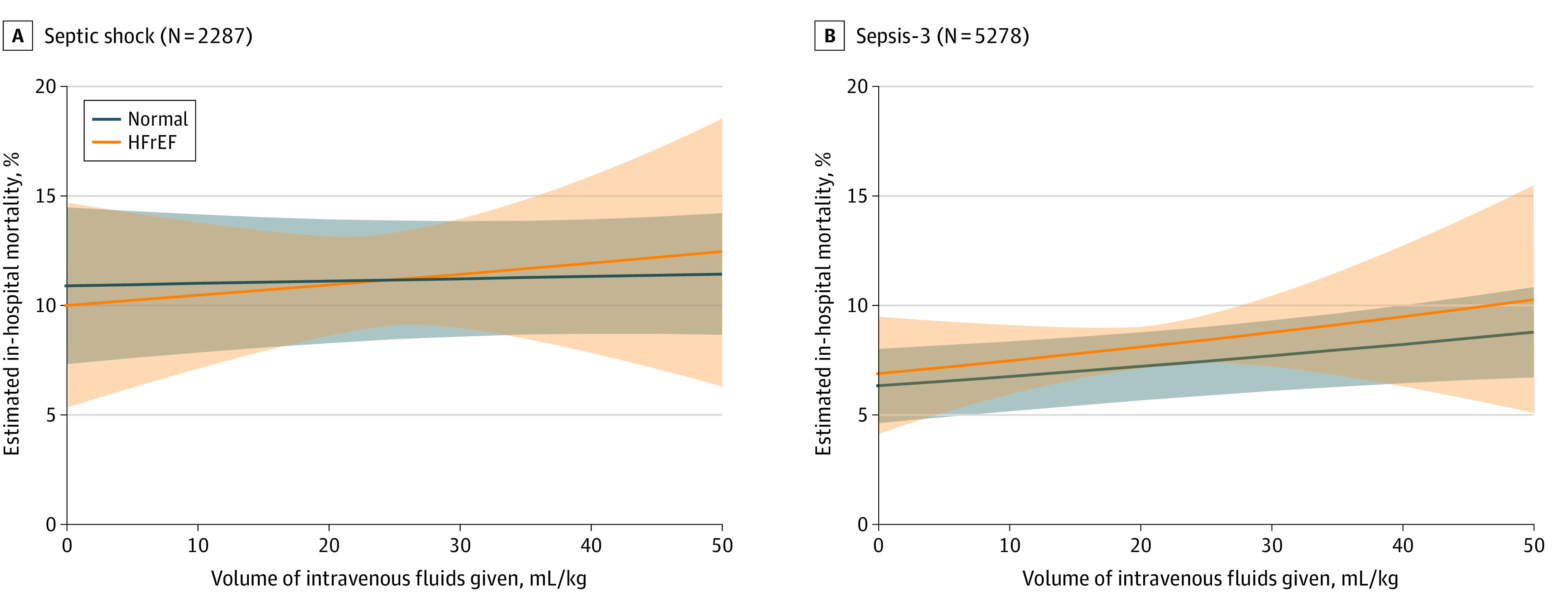
Estimated In-Hospital Mortality From Multivariable Models Adjusted risk of estimated in-hospital mortality by volume of intravenous fluids administered in the first 6 hours. Shaded areas indicate 95% CIs. HFrEF indicates heart failure with reduced ejection fraction.

After multivariable adjustment for potential confounders, preexisting HFrEF was associated with a reduced risk-adjusted odds of receiving 30 mL/kg of intravenous fluids within the first 6 hours among patients with septic shock (aOR, 0.63; 95% CI, 0.47-0.85; *P* = .002) (Table 2; Figure 2) compared with those without HFrEF. Higher presenting SOFA score (aOR, 1.09; 95% CI, 1.01-1.18; *P* = .03), lower age (aOR, 0.98; 95% CI, 0.98-0.99; *P* < .001), lower Elixhauser Comorbidity Index (aOR, 0.91; 95% CI, 0.87-0.95; *P* < .001), and female sex (aOR for male sex, 0.71; 95% CI, 0.58-0.87; *P* = .001) were also associated with increased odds of receiving guideline-recommended intravenous fluids. For secondary outcomes, HFrEF was not associated with greater in-hospital mortality (aOR, 0.92; 95% CI, 0.69-1.24; *P* = .59) (eTable 2 in the Supplement; Figure 3), and there was no interaction between HFrEF and the volume of intravenous fluid administered within the first 6 hours (Wald test of interaction; aOR, 1.00; 95% CI, 0.98-1.03; *P* = .72; Figure 3).

**Table 2.** Multivariable Model of Receipt of 30 mL/kg of Intravenous Fluids Within 6 Hours of Septic Shock Onset (N = 2287)

Variable^a^	aOR (95% CI)	*P* value
HFrEF	0.63 (0.47-0.85)	.002
Age^b^	0.98 (0.98-0.99)	<.001
Race		
Black	1 [Reference]	NA
White	0.87 (0.69-1.10)	.87
Other^c^	0.95 (0.52-1.72)	.26
Sex		
Female	1 [Reference]	NA
Male	0.71 (0.58-0.87)	.001
Elixhauser Comorbidity Index^d^	0.91 (0.87-0.95)	<.001
SOFA score in 6 h^e^	1.09 (1.01-1.18)	.03

Abbreviations: aOR, adjusted odds ratio; HFrEF, heart failure with reduced ejection fraction; SOFA, Sequential Organ Failure Assessment.

^a^ Hospital of admission included as a random effect.

^b^ The aOR corresponds to a 1-year increase in age.

^c^ American Indian or Alaska Native, Asian, Chinese, Filipino, Hawaiian or Other Pacific Islander, Middle Eastern, Native American, or Pacific Islander.

^d^ A method of categorizing the comorbid conditions of patients based on the *International Classification of Diseases, Ninth Revision* diagnosis codes found in administrative data (range, 0-31; with higher score indicating greater comorbidity burden). The aOR corresponds to a 1-point change in the Elixhauser Comorbidity Index.

^e^ Corresponds to the severity of organ dysfunction, reflecting 6 organ systems (cardiovascular, hepatic, hematologic, respiratory, neurologic, and kidney), each with a score range of 0 to 4 points, with a total score range of 0 to 24 points. The aOR corresponds to a 1-point increase in SOFA score.

### Sensitivity Analyses

Model results were robust to sensitivity analysis. First, the E-value indicated that unmeasured confounders would require an aOR of at least 1.83 (lower limit of 95% CI, 1.39) to negate the association between HFrEF and administration of 30 mL/kg of intravenous fluids. Second, when the primary model included all patients meeting Sepsis-3 criteria, preexisting HFrEF remained associated with decreased odds of receiving 30 mL/kg of intravenous fluids (aOR, 0.64; 95% CI, 0.48-0.87; *P* = .004) (eTable 3 in the Supplement; Figure 2). When we included all patients with Sepsis-3 criteria in mortality models, preexisting HFrEF still had no association with in-hospital mortality (aOR, 1.00; 95% CI, 0.82-1.22; *P* = .99) (eTable 4 in the Supplement; Figure 3). Third, when patients with transthoracic echocardiograms obtained within 1 month of incident sepsis were excluded (n = 814), models for fluid administration (aOR, 0.68; 95% CI, 0.51-0.90; *P* = .008) and in-hospital mortality (aOR, 1.08; 95% CI, 0.83-1.41; *P* = .56) were consistent. Fourth, the addition of RV dysfunction as a potential confounder did not change the association between HFrEF and fluid administration (aOR, 0.69; 95% CI, 0.50-0.96; *P* = .03) or mortality (aOR, 0.87; 95% CI, 0.60-1.26; *P* = .45). Fifth, with the use of continuous LVEF as the exposure of interest, a 10-point increase in LVEF was associated with increased odds of receiving 30 mL/kg of intravenous fluids (aOR, 1.17; 95% CI, 1.09-1.26; *P* < .001; Figure 2) but was not associated with mortality (aOR, 1.02; 95% CI, 0.96-1.07; *P* = .57).

## Discussion

In an integrated multicenter health care system of community and academic hospitals, preexisting HFrEF was common and associated with a reduced risk-adjusted odds of receiving guideline-recommended intravenous fluids among patients with community-acquired septic shock. Heart failure with reduced ejection fraction was not associated with in-hospital mortality, and intravenous fluid administration did not modify the association between preexisting HFrEF and outcome.

Aggressive, early intravenous fluid administration for septic shock is controversial. Some observational studies show a potential benefit associated with the administration of fluids early in sepsis.^25,26^ However, to our knowledge, no randomized clinical trials demonstrate a specific fluid volume or resuscitation strategy that improves outcomes.^25,27,28,29^ Furthermore, excess fluid administration can lead to adverse outcomes, including mechanical ventilation and mortality.^27,30^ Patients with preexisting HFrEF are a particularly challenging subgroup of patients with sepsis, for whom the evidence is even more inconsistent.^13,14,15,31^ Our study expands on existing knowledge by showing that patients with preexisting HFrEF are less likely to receive guideline-recommended intravenous fluids, despite showing no differences in markers of illness severity or completion rates of other guideline-recommended care (eg, prompt antibiotics and measurement of serum lactate level).

These results also show that differences in fluid administration practice patterns are not associated with different clinical outcomes, even among patients with preexisting HFrEF. Prior studies have reached variable conclusions about the association of HFrEF with mortality among patients with sepsis, with some studies showing no association and others showing increased mortality among patients with HFrEF.^10,12,13,14,15,16,17,18,31,32^ One potential explanation for these variable findings is the inconsistency in how heart failure is defined. Many groups rely on administrative billing codes to define HFrEF; these codes may have poor sensitivity and positive predictive value.^33^ To more accurately classify patients, we included only patients with a transthoracic echocardiogram within 1 year of incident sepsis to characterize the presence or absence of preexisting HFrEF by the AHA definition.^22^ In addition, our echocardiographic data included other cardiac traits, such as RV and valvular dysfunction.

Clinical equipoise exists surrounding the appropriate management of patients with sepsis and preexisting HFrEF. Physicians likely administer less fluid for these patients owing to concerns for adverse outcomes, such as increased pulmonary edema, respiratory failure, and mortality. Nonetheless, our study found that HFrEF was not associated with increased in-hospital mortality and that the volume of fluid administered does not modify this association. One possible explanation is that, while fluid administration differed, the true treatment effect of intravenous fluid is small relative to other interventions. A focus on timely administration of appropriate antibiotics may be more critical.^34^ In addition, the use of point-of-care ultrasonography in making clinical assessments of volume status may augment clinical decision-making. It is also possible that our findings were underpowered to detect a meaningful difference in outcome between groups.

### Limitations

This study has some limitations. First, as with all observational studies, unmeasured confounders may be present. These additional confounders are unlikely to alter the study conclusions, however, as the E-value was larger than the effect size of any of the typical measured confounding variables. Second, clinical practice guidelines for intravenous fluid administration in sepsis were evolving during our study period. The Surviving Sepsis Campaign has recommended a 30-mL/kg intravenous fluid bolus for sepsis-induced hypoperfusion since the 2012 guidelines,^35^ but the CMS SEP-1 performance metric was not implemented until the end of our study period. Although the changes in clinician practice after the implementation of the CMS SEP-1 metric were modest,^36^ the effect on patients with HFrEF is unknown. Third, the health care system we studied provides care in a specific geographic region, and the external validity of these results may be different outside the US. Fourth, we used transthoracic echocardiogram data obtained within 1 year of sepsis hospitalization, but these data may not account for the temporal changes in systolic function during that time frame. Fifth, the targets (eg, bedside assessment of volume status and volume responsiveness) guiding a clinician’s resuscitation decisions for each patient may not be fully represented by the clinical data available in the electronic health record. Sixth, we used the AHA criteria to define HFrEF based on a binary EF cutoff. However, many other factors may play a role in patient outcomes, such as the trajectory of EF changes, the cause of heart failure, and the presence of diastolic dysfunction. Given potential inaccuracies in quantifying these additional factors using electronic health record data alone, we have not examined these features in this study.

## Conclusions

The results of this cohort study of patients with community-acquired septic shock suggest that preexisting HFrEF was common and associated with reduced odds of receiving guideline-recommended intravenous fluids. However, HFrEF was not associated with in-hospital mortality, and intravenous fluid administration did not modify the association between HFrEF and outcome.

## Notes

Supplement. **eFigure.** Frequency Distribution of Left Ventricular Ejection Fraction in Patients With Sepsis-3 and Septic Shock **eTable 1**. Crude Outcomes **eTable 2.** Multivariable Model of In-Hospital Mortality Among Patients With Septic Shock (N=2,287) **eTable 3.** Sensitivity Analyses – 30 mL/kg Intravenous Fluid Administration **eTable 4.** Sensitivity Analyses – In-Hospital Mortality
