# Supplementary material for: Association Between Preexisting Heart Failure With Reduced Ejection Fraction and Fluid Administration Among Patients With Sepsis
Source: JAMA Netw Open. 2022 Oct 7;5(10):e2235331. doi: 10.1001/jamanetworkopen.2022.35331 (PMC9547322; doi:10.1001/jamanetworkopen.2022.35331)
Supplement: Supplement. — eFigure. Frequency Distribution of Left Ventricular Ejection Fraction in Patients With Sepsis-3 and Septic Shock eTable 1. Crude Outcomes eTable 2. Multivariable Model of In-Hospital Mortality Among Patients With Septic Shock (N=2,287) eTable 3. Sensitivity Analyses – 30 mL/kg Intravenous Fluid Administration eTable 4. Sensitivity Analyses – In-Hospital Mortality [file jamanetwopen-e2235331-s001.pdf]

Supplementary Online Content

Powell RE, Kennedy JN, Senussi MH, Barbash IJ, Seymour CW. Association between preexisting heart failure with reduced ejection fraction and fluid administration among patients with sepsis. *JAMA Netw Open.* 2022;5(10):e2235331. doi:10.1001/jamanetworkopen.2022.35331

eFigure. Frequency Distribution of Left Ventricular Ejection Fraction in Patients With Sepsis-3 and Septic Shock
eTable 1. Crude Outcomes
eTable 2. Multivariable Model of In-Hospital Mortality Among Patients With Septic Shock (N=2,287)
eTable 3. Sensitivity Analyses – 30 mL/kg Intravenous Fluid Administration
eTable 4. Sensitivity Analyses – In-Hospital Mortality

This supplementary material has been provided by the authors to give readers additional information about their work.

eFigure. Frequency Distribution of Left Ventricular Ejection Fraction in Patients With Sepsis-3 and Septic Shock

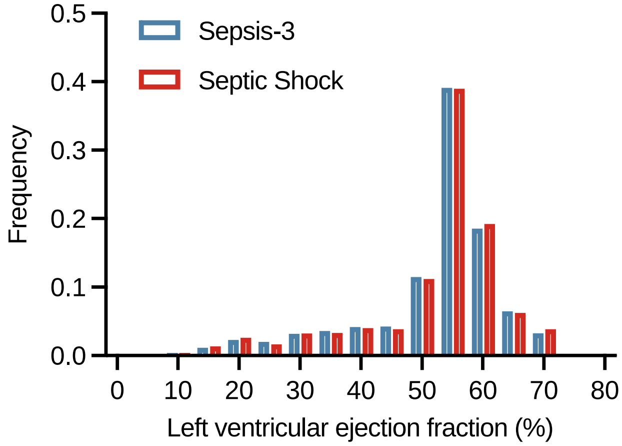

Frequency distribution of left ventricular ejection fraction among patients with Sepsis-3 (blue) and septic shock (red).

© 2022 Powell RE et al. *JAMA Network Open.*

eTable 1. Crude Outcomes

	Septic Shock		Sepsis-3	
**Outcomes**	**Normal EF (N = 1,911)**	**HFrEF (N = 380)**	**Normal EF (N = 4,394)**	**HFrEF (N=884)**
In-hospital mortality – no. (%)	244 (12.8)	47 (12.4)	371 (8.4)	78 (8.8)
Mechanical ventilation – no. (%) [a]	549 (28.7)	96 (25.3)	883 (20.1)	143 (16.2)
Total days – median (IQR)	5 (3, 10)	4 (3, 10)	5 (2, 9)	4 (2, 9.5)
Vasopressors – no. (%) [b]	591 (30.9)	121 (31.8)	591 (13.5)	121 (13.7)
Total days – median (IQR)	3 (2, 4)	3 (2, 5)	3 (2, 4)	3 (2, 6)
ICU admission – no. (%)	1495 (78.2)	304 (80.0)	2565 (58.4)	514 (58.1)
Hospital length of stay – median (IQR), days	7.1 (4.5, 11.5)	6.9 (4.5, 11.5)	6.2 (3.9, 10.1)	6.2 (3.9, 9.9)
Intravenous fluid volume given in 6 hours – median (IQR), mL/kg [c]	20.5 (7.7, 39.4)	15.6 (5.3, 30.2)	10.1 (1.6, 24.6)	6.1 (0.9, 18.9)
30 cc/kg given in 6 hours – no. (%)	699 (36.6)	96 (25.3)	885 (20.1)	113 (12.8)

[a] Includes invasive mechanical ventilation delivered at any time during the hospitalization, either via endotracheal tube or tracheostomy, excluding noninvasive ventilation strategies

[b] Vasopressors include norepinephrine, epinephrine, phenylephrine, vasopressin, and dopamine

[c] Intravenous fluid volume is calculated based on ideal body weight, and represents all crystalloid solution given within the first 6 hours of sepsis onset.

Abbreviations: EF: ejection fraction; HFrEF: heart failure with reduced ejection fraction; IQR: interquartile range; ICU: intensive care unit

© 2022 Powell RE et al. *JAMA Network Open.*

**eTable 2.** Multivariable Model of In-Hospital Mortality Among Patients With Septic Shock (N=2,287)			
**Variable [a]**	**aOR**	**aOR 95% CI**	***P*-value**
HFrEF	0.92	0.69 – 1.24	0.590
Age [b]	1.03	1.02 – 1.04	<0.001
Race			
Black	REF	-	-
White	0.67	0.51 – 0.90	0.007
Other [c]	1.52	0.76 – 3.05	0.241
Sex			
Female	REF	-	-
Male	1.03	0.76 – 1.37	0.890
Elixhauser [d]	0.99	0.92 – 1.06	0.722
SOFA in 6 hours [e]	1.26	1.20 – 1.32	<0.001

[a] Hospital of admission included as a random effect
[b] Adjusted OR corresponds to a 1 year increase in age.
[c] Other race corresponds to Chinese, Filipino, Hawaiian, American Indian/Alaskan, Asian, Hawaiian/Other Pacific Islander, Middle Eastern, Native American, or Pacific Islander
[d] Elixhauser is a method of categorizing comorbidities of patients based on the International Classification of Diseases (ICD) diagnosis codes found in administrative data, ranging from 0 to 31. Adjusted OR corresponds to a 1 point change in Elixhauser.
[e] SOFA score corresponds to the severity of organ dysfunction, reflecting six organ systems each with a score range of 0 to 4 points (cardiovascular, hepatic, hematologic, respiratory, neurological, renal), with a total score range of 0 to 24 points. Adjusted OR corresponds to a 1 point change in SOFA score.
Abbreviations: aOR: adjusted odds ratio; CI: confidence interval; HFrEF: heart failure with reduced ejection fraction; SOFA: sequential organ failure assessment score

© 2022 Powell RE et al. *JAMA Network Open.*

**eTable 3.** Sensitivity Analyses – 30 mL/kg Intravenous Fluid Administration			
**Feature [a]**	**aOR - HFrEF**	**aOR 95% CI**	**P-value**
Primary Model	0.63	0.47 – 0.85	0.002
Including all patients with Sepsis-3	0.64	0.48 – 0.87	0.004
Excluding TTEs obtained within 1 month of sepsis	0.68	0.51 – 0.90	0.008
Including RV dysfunction as a covariate	0.69	0.50 – 0.96	0.028

[a] Hospital of admission included as random effect
Abbreviations: aOR: adjusted odds ratio; CI: confidence interval; HFrEF: heart failure with reduced ejection fraction; TTE: transthoracic echocardiogram; RV: right ventricular

© 2022 Powell RE et al. *JAMA Network Open.*

**eTable 4.** Sensitivity Analyses – In-Hospital Mortality			
**Feature [a]**	**aOR - HFrEF**	**aOR 95% CI**	**P-value**
Primary Model			
Including all patients with Sepsis-3	1.00	0.82 – 1.22	0.993
Excluding TTEs obtained within 1 month of sepsis	1.08	0.83 – 1.41	0.561
Including RV dysfunction as a covariate	0.87	0.60 – 1.26	0.449

[a] Hospital of admission included as random effect
Abbreviations: aOR: adjusted odds ratio; CI: confidence interval; HFrEF: heart failure with reduced ejection fraction; TTE: transthoracic echocardiogram; RV: right ventricular

© 2022 Powell RE et al. *JAMA Network Open.*
